# The Role of Probiotics in Healing Burns and Skin Wounds; An Integrative Approach in the Context of Regenerative Medicine

**DOI:** 10.3390/life15091434

**Published:** 2025-09-12

**Authors:** Lenuta Ambrose, Ciprian Adrian Dinu, Gabriela Gurau, Nicoleta-Maricica Maftei, Madalina Nicoleta Matei, Maria-Andrada Hincu, Marius Radu, Mihaela-Cezarina Mehedinti

**Affiliations:** 1 Faculty of Medicine and Pharmacy, “Dunarea de Jos” University of Galati, 800008 Galati, Romania; lenuta.ambrose@ugal.ro (L.A.); c.dinu@ugal.ro (C.A.D.); gabriela.gurau@ugal.ro (G.G.); madalina.matei@ugal.ro (M.N.M.); mihaela.mehedinti@ugal.ro (M.-C.M.); 2 Research Centre in the Medical-Pharmaceutical Field, “Dunarea de Jos” University of Galati, 800010 Galati, Romania; 3 Emergency Clinical Hospital for Children “Sf Ioan”, 800487 Galati, Romania; 4 Department of Pharmaceutical Sciences, Faculty of Medicine, and Pharmacy, “Dunarea de Jos” University of Galati, 800010 Galati, Romania; 5 Faculty of Biology, University Ovidius of Constanta, 900470 Constanta, Romania; drdmaryus@yahoo.com

**Keywords:** thermal burns, probiotics, immunomodulation, eubiosis, postbiotics, angiogenesis

## Abstract

In the context of thermal injury, local tissue integrity and systemic homeostasis are compromised, often resulting in delayed healing, infections, and disturbances of the skin and intestinal microbial balance. Despite several reviews addressing probiotics in wound healing, none has specifically focused on their role in thermal injuries and burn-associated pathophysiology. This review uniquely integrates evidence on the gut–skin axis, postbiotic innovations, and regenerative perspectives tailored to burn care. We conducted a critical synthesis of recent preclinical and clinical trials evaluating the use of probiotics and their derivatives to promote tissue regeneration following burn injury. Previous reviews have addressed probiotics in general wound repair, but the present synthesis advances the field by bridging mechanistic insights (immune modulation, angiogenesis, microbiome restoration) with translational evidence in burn patients, offering a framework for personalized regenerative approaches. Based on a structured review of the literature—including in vitro models, animal experiments, and randomized trials with topical, enteral, and systemic administration of probiotic—we identified four main mechanisms of action: modulation of the immune response by balancing cytokines and polarization of T lymphocytes; stimulation of tissue repair by increasing the proliferation of keratinocytes and fibroblasts, increased collagen synthesis, and induction of angiogenesis; direct antimicrobial activity against biofilms and multiresistant pathogens; and the restoration of eubiosis with the improvement of the function of epithelial barriers. While these findings endorse the adjunctive use of probiotics in burn management, large multicenter trials are required to standardize strains, dosages, and formulations before their routine clinical adoption.

## 1. Introduction

Skin burns are severe traumatic injuries to the skin and/or underlying tissues caused by the action of thermal, chemical, electrical, or radiation agents. They induce a complex local and systemic dysfunction with major implications on the integrity of the skin barrier, the body’s homeostasis and immune reactivity. Depending on the severity and extent of the lesion, burns can lead to serious complications including systemic infections, hypovolemic shock, multiorgan dysfunction, and even death [1]. The healing process of burn wounds is much more complex and slow compared to other types of skin lesions, as it involves both regeneration of the epidermis and restoration of deep dermal structures. The classical stages of healing—inflammation, proliferation, and remodeling—are often altered by the presence of infections, the deficient nutritional status of the burned patient, and, finally, by imbalances in the microbiome. Dysbiosis of the intestine and skin, caused by antibiotics, excessive inflammatory response, and loss of barrier function, dramatically affects mucosal and systemic immunity, prolonging the inflammatory phase and delaying reepithelization [2]. In this context, modern medicine is exploring new therapeutic adjuvants to complement conventional surgical and pharmacological interventions. This review provides a novel integrative perspective by linking mechanistic pathways with clinical outcomes in burn care. Probiotics, defined as living microorganisms that when taken in appropriate doses confer health benefits to the host [3], have become the subject of increased scientific interest in their influence on the healing of acute and chronic wounds [4]. They exert anti-inflammatory, immunomodulating, antimicrobial, and regenerative effects, being able to intervene in multiple stages of tissue healing [5,6]. Moreover, the role of probiotics is not limited to maintaining intestinal eubiosis. Recent studies suggest that certain strains can be applied topically, in the form of gels, biofilms, or impregnated dressings, demonstrating efficiency in accelerating reepithelization, reducing bacterial colonization, and supporting angiogenesis [7]. Probiotics such as *Lactobacillus rhamnosus* and *Saccharomyces boulardii* have been tested with promising results in preclinical models and pilot clinical trials [8,9]. Also, the concept of “postbiotics”, bioactive compounds resulting from the metabolic activity of probiotics (e.g., peptides, short-chain fatty acids, exopolysaccharides), opened new therapeutic directions in dermatology and plastic surgery, giving the advantage of an increased safety profile compared to living microorganisms [10,11].

These substances can reduce oxidative stress, modulate the expression of genes involved in tissue regeneration, and induce local immunological tolerance [12]. The integration of probiotics in the treatment of burns aligns with current trends in regenerative medicine, which are aimed at biological, personalized, and minimally invasive therapies. At the same time, in the context of the global threat of antimicrobial resistance, the use of probiotics as alternative or complementary agents of classical antimicrobials is considered a strategic direction in the management of post-burn infections [13,14,15]. The aim of this review is to deliver a comprehensive and critical synthesis of current evidence on probiotics and their derivatives in burn care, spanning molecular mechanisms to clinical outcomes. Unlike prior overviews, it emphasizes the distinctive pathophysiological context of burns, integrating insights on the gut–skin axis, next-generation probiotics, and postbiotic strategies. By identifying knowledge gaps and outlining future research priorities, this work provides a timely framework to advance the integration of probiotics into modern regenerative medicine.

## 2. Methods

### 2.1. Search Strategy and Data Sources

A comprehensive literature search was systematically performed in the major biomedical databases, including PubMed/MEDLINE, Scopus, Web of Science, and Cochrane Library, to capture both preclinical and clinical studies relevant to the topic. The search covered publications published between January 2000 and July 2025, thereby encompassing more than two decades of research on the role of probiotics in burn care and wound healing.

The search strategy was constructed using a combination of free-text terms and controlled vocabulary (MeSH/Emtree) to maximize sensitivity. The primary concepts combined were “burn injury” OR “thermal injury” OR “skin wound” (representing the clinical condition) AND “probiotics” OR “synbiotics” OR “postbiotics” OR “next-generation probiotics” (representing the interventions of interest). Boolean operators (AND, OR) and truncation was employed to ensure retrieval of all relevant variations and related terms. In addition to database queries, the reference lists of key systematic reviews, narrative overviews, and seminal original studies were screened manually to identify further eligible publications not captured through electronic search. Gray literature, conference proceedings, and unpublished data were not considered in this review.

### 2.2. Eligibility Criteria and Assessment of Evidence

We included both preclinical studies (in vitro and animal models) and clinical investigations (observational studies, pilot trials, randomized controlled trials, and meta-analyses) that evaluated probiotics or their derivatives in burn wound healing and post-burn infections. Exclusion criteria were articles not written in English, conference abstracts without full text, case reports, and editorials. Clinical and preclinical studies were critically appraised for methodological clarity, completeness of reporting, and relevance to burn care.

**Figure 1:**
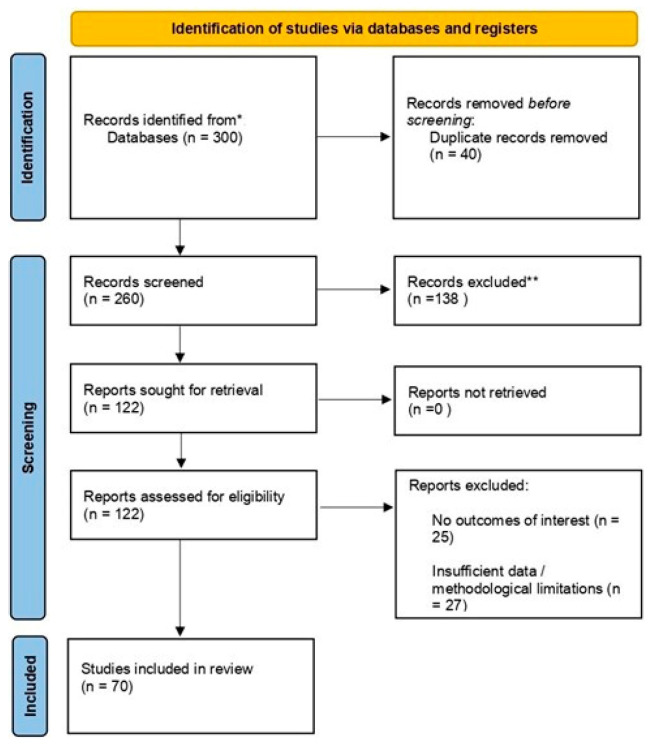
PRISMA 2020 flow diagram of the study selection process.* = 300, ** = 138.

Particular attention was given to study design, sample size, reproducibility of results, and consistency across different interventions. The initial database search identified approximately 300 records. After removal of duplicates, titles and abstracts were screened for relevance. Full texts were then assessed against inclusion and exclusion criteria. The selection process is illustrated in a PRISMA 2020 flow diagram (Figure 1).

## 3. Epidemiological Aspects

Burn injuries are a significant cause of morbidity and mortality worldwide, having a considerable impact on public health, the economy of medical systems, and the quality of life of affected patients. In modern classifications, burns are considered major trauma, equivalent to multisystemic injuries, due to severe local and systemic implications, including generalized inflammatory response, immunological imbalance, massive oxidative stress, infections, and organic dysfunction. The prevalence of this type of trauma, accompanied by its socioeconomic and therapeutic burden, justifies international efforts aimed at optimizing prevention, treatment, and post-burning recovery. According to the World Health Organization (WHO), more than 11 million people are affected annually by burns requiring medical treatment, and about 180,000 deaths are recorded annually, especially in low- and middle-income countries [16]. Mortality is particularly high in the regions of Southeast Asia and Sub-Saharan Africa, where medical infrastructure is poorly developed and access to intensive care units or specialist centers for burned patients is limited or non-existent. These statistics are more alarming as many of the serious burns are completely preventable, caused by uncontrolled exposure to open fire sources, volatile fuels, electricity, or caustic chemicals. In vulnerable populations, children under 5 years of age and the elderly are the most affected. In the case of children, burns are generally accidental and occur predominantly in the domestic environment, through contact with hot liquids, barbecues, hobs, or radiators [17]. In contrast, in older people, the appearance of burns is favored by comorbidities, reduced mobility, delayed reflexes, visual impairments, and lack of functional autonomy. Thus, the impact on these categories is disproportionate, resulting in increased rates of mortality and severe disability.

In developed countries such as those in the European Union, the United States, and Canada, the incidence of burns is lower, and mortality has decreased significantly over the past two decades due to advances in intensive management, infection control, clinical nutrition, and regenerative therapies [18]. For example, a European multicenter study [19] estimated an average incidence of burn hospitalizations between 120 and 150 cases per 100,000 inhabitants annually, with an average mortality below 4%. However, complex cases—with extensive impairment, respiratory burns, or deep burns in functional regions—remain associated with significant mortality [20]. In Romania, national statistics indicate an increased incidence of moderate and severe burns, especially in rural areas and among the low-income population. According to the National Institute of Statistics (2022) [21], over 6000 burn cases require hospitalization annually, and about 800–1200 of them are treated in intensive care units. In the absence of an integrated national system for the rapid transfer of burned patients and a network of specialized regional centers, Romania frequently faces delays in treatment, serious nosocomial infections, and overmortality [21]. From an etiological perspective, the most common causes of burns vary geographically. Globally, thermal burns (hot liquids, open fire) account for over 80% of cases. In the industrial environment, electrical and chemical burns are more common, associated with deep injuries and severe systemic complications, including rhabdomyolysis and compartment syndrome. In urban centers of developed countries, cases of burns caused by lithium-ion battery explosions, pyrotechnic incidents, and laboratory accidents have been reported in recent years, indicating a change in the epidemiological profile with technological evolution [22]. Another major issue is the economic impact generated by burned patient care. Direct costs—including prolonged hospitalization, reconstructive surgery, advanced dressings, antimicrobial therapies, and parenteral nutrition—may exceed EUR 200,000 per severe case in Western countries [23]. This includes indirect costs such as loss of productivity, social compensation, psychological rehabilitation, and reintegration into employment, which have a profound economic impact, particularly on families and communities with limited resources. The COVID-19 pandemic has brought additional challenges in the treatment of burn patients. The reorientation of medical resources to SARS-CoV-2-infected patients has led to delayed admissions, reduced access to reconstructive surgery, and prolonged exposure of burned patients to resistant nosocomial infections. Restrictions on interregional transfers and lack of personal protective equipment also negatively influenced treatment and recovery in cases of critical burn. In this complex and dynamic epidemiological context, the emergence of adjuvant, effective, and safe therapeutic strategies is of great importance. Probiotics, through their effects on intestinal homeostasis supporting mucosal immunity, reducing bacterial translocation, and modulating systemic inflammatory response, can play a relevant role in decreasing the incidence of infections and accelerating tissue healing in burned patients. Recent epidemiological data provide biological and clinical justification for exploring these biotherapeutic interventions, particularly in contexts where infection control is a major challenge [23].

## 4. The Pathophysiology of Burns and Tissue Healing

Burns and tissue healing pathophysiology represent a distinctive category of trauma—thermal, chemical, electrical, or radiative—that causes both local, structural, and functional skin damage and a complex systemic response, often disproportionate to the extent of the injury. Depending on depth and surface area, burns can trigger intense local inflammation, systemic inflammatory response syndrome (SIRS), immune dysregulation, multiorgan dysfunction, and, in severe cases, death [24,25]. At the systemic level, disruption of the skin barrier leads to fluid loss, hypovolemia, and the release of inflammatory mediators that promote vascular leakage, tissue edema, and impaired perfusion to vital organs. These effects often lead to burn shock, especially in major burns involving more than 20% of the total body surface area [24,25]. Understanding the pathophysiological mechanisms of burns—ranging from cytokine storms and oxidative stress to microbiome alterations—is crucial for guiding clinical interventions such as early fluid resuscitation, infection control, and targeted immune modulation.

### 4.1. Local Burn Response

Locally, a burn destroys epidermal and dermal cells, disrupts the collagen network and dermal micro-vasculature, and provokes the immediate release of inflammatory mediators such as IL-1, IL-6, prostaglandins, and histamine. These mediators drive vasodilation, raise capillary permeability, and recruit polymorphonuclear leukocytes to the wound bed [26,27]. According to Jackson’s classic three-zone concept (coagulation, stasis, hyperaemia), early supportive care in the first 48–72 h is critical to halt progression of necrosis and salvage marginally viable tissue [27].

### 4.2. Systemic Response

Extensive injuries (involving 20–30% of body surface area) instigate an intense systemic response characterized by the release of pro-inflammatory cytokines into circulation, the onset of a hypermetabolic syndrome, and increased risk of multiorgan dysfunction [28]. Levels of catecholamines, cortisol, and glucagon rise sharply, leading to hyperglycemia, protein catabolism, nitrogen loss, and insulin resistance [24]. Systemic capillary hyperpermeability causes plasma extravasation into interstitial tissues, resulting in massive edema and hypovolemia; without timely fluid resuscitation, burn shock ensues [28]. Additionally, severe burns compromise intestinal barrier integrity, facilitating bacterial translocation and increasing the risk of sepsis [29].

### 4.3. Imbalances of Immunity in Burns

Severe thermal trauma induces profound suppression of both cellular and humoral immunity: dendritic cell, helper T-cell, and NK cell activity declines, while neutrophil overactivation promotes free radical production and collateral tissue damage. Elevated IL-10 contributes to post-traumatic immunosuppression. This dysfunction underlies the heightened vulnerability of burn patients to nosocomial infections and sepsis, particularly by antibiotic-resistant pathogens such as *Pseudomonas aeruginosa*, *Acinetobacter baumnnii*, and methicillin-resistant *Staphylococcus aureus* (MRSA) [30].

### 4.4. The Healing Process of Burn Wounds

The healing process of a burned wound follows broadly the same steps as other tissue damage but is often disturbed by the deep and contaminated nature of the injury [1]. The three main phases are as follows: Inflammatory phase (0–5 days): characterized by vasodilation, extravasation of plasma, recruitment of macrophages and neutrophils, as well as degradation of necrotic tissue [31]. Proliferative phase (5–20 days): includes angiogenesis, fibroblast migration, type III collagen synthesis, and temporary extracellular matrix formation [31]. Remodeling phase (up to 12 months): involves the conversion of type III collagen into type I collagen, reorganization of dermal architecture, and scar maturation [1,31,32].

In case of deep burns, this process is severely affected by the persistence of infection, tissue oxygen deficiency, chronic inflammation, and nutritional imbalances [1,32,33]. In addition, abnormal scarring (hypertrophic or keloid) is common, with severe functional and aesthetic impact.

### 4.5. The Role of Microbiome and the Impact on Regeneration

A newly investigated aspect is the influence of the intestinal and cutaneous microbiome on the post-burn response. Thermal trauma induces profound intestinal dysbiosis, characterized by the loss of beneficial commensal species such as *Lactobacillus* and *Bifidobacterium*, and the concurrent overgrowth of pathogenic bacteria including *Enterobacteriaceae*, which contributes to bacterial translocation and systemic inflammation [31]. Disruption of the skin microbiome similarly impairs the skin’s ability to synthesize antimicrobial peptides and maintain barrier integrity [2]. In this context, the use of probiotics and postbiotics has emerged as a promising strategy to restore microbial homeostasis, attenuate inflammation, and enhance re-epithelialization in burn wounds.

## 5. Classification of Burns and Conventional Therapeutic Approach

Skin burns are traumatic injuries of considerable pathological complexity, involving partial or total degradation of the skin structure and, in severe cases, the underlying tissues. Effective management of burn patients relies heavily on accurate and standardized classification systems which are essential to assess clinical severity, predict complications, and plan appropriate interventions. The classification of burns encompasses several key pathophysiological parameters, including the depth of tissue damage, the extent of affected total body surface area (TBSA), the etiology of the burn (e.g., thermal, electrical, chemical), the anatomical location, and the presence of comorbidities or systemic risk factors such as inhalation injury, advanced age, or sepsis risk.

### 5.1. Classification According to the Depth of the Lesion [33]

**Figure 2:**
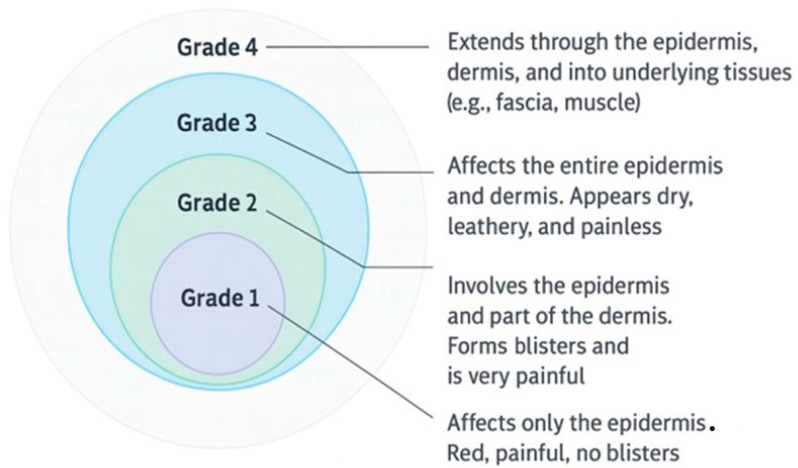
Classification of burn injuries by grade, showing the depth of tissue damage and associated clinical features from superficial (Grade 1) to full-thickness and deep tissue involvement (Grade 4).

The depth of the burn is determined by the intensity and duration of exposure to the causative agent, as well as the individual characteristics of the patient (age, thickness of the skin, vascular comorbidities). The standard histological classification includes four degrees (Figure 2):

- Grade I (superficial) burns: Affect exclusively the epidermal layer. Clinically, it is manifested by erythema, pain, and increased sensitivity to touch, without the formation of blisters. Healing occurs spontaneously in 3–6 days, without scarring, by rapid regeneration of basal keratinocytes. They are typical for excessive exposure to UV radiation (e.g., sunburn).
- Grade II burns (partially thick): Grade II superficial: Involve the epidermis and superficial portion of the papillary dermis. It is characterized by vesicles, intense erythema, and severe exudate pain. Healing occurs in 10–14 days, with complete restoration of the dermo–epidermal structure and minimal risk of scarring. Degree II deep: Affect up to the deep reticular dermis. The lesion areas are pale or marbled, the pain is reduced (due to damage to the nerve endings), and spontaneous healing is unlikely. In most cases, excision and covering with a cutaneous graft is required.
- Grade III burns (total thickness): Involve the complete destruction of the epidermis, dermis, and cutaneous appendages (pile follicles, sebaceous glands, sweat glands). The skin appears dry, white, brown, or charred and is insensitive to touch. Healing without surgery is impossible. Autologous cutaneous grafting is the therapeutic standard.
- Grade IV burns: Exceed the dermo–epidermal plane and affect the subcutaneous structures: fascia, muscle, tendons, bone. They are common in deep or explosive electrical injuries. The functional prognosis is severe, frequently requiring amputations or complex reconstructions.

### 5.2. Estimation of the Burn Extension

The correct determination of the burned surface is an essential element in determining the need for fluids, in prognostic, and in choosing surgical treatment. The most used methods are as follows: Wallace’s Rule (“Rule of 9”): Divide the body area into percentage regions: head—9%; anterior thorax—18%; posterior thorax—18%; each upper member—9%; each lower member—18% [34]. This method is fast and practical, but less accurate in children and people with atypical conformation. The Lund-Browder scheme: Provides a precise, age-adjusted estimate, being the gold standard in pediatrics and research. TBSA 10% in children or 15–20% in adults are considered clinically significant and justifies volume resuscitation, hospitalization, and multidisciplinary intervention [35].

### 5.3. Topographic and Functional Classification

Certain anatomical areas are considered critical due to their high risk for severe functional or aesthetic sequelae:

- Face—risk of deformity, microstomia, and ocular injury;
- Hands—high potential for joint contractures and loss of function;
- Perineum and genitals—increased infection risk, particularly challenging area to manage;
- Neck/throat region—prone to contracture formation that may impair airway and respiratory function;
- Large joints—burns involving biomechanical joints such as elbows, knees, or shoulders can lead to long-term functional impairment;
- Deep or extensive burns in these regions often necessitate early surgical excision and timely rehabilitation to preserve function and reduce aesthetic damage.

### 5.4. Conventional Therapeutic Approach

Burn management is organized in a stepwise fashion, tailored to burn depth, TBSA, anatomical location, and patient condition.

#### 5.4.1. Minor Burns (Grade I and Superficial Grade II; TBSA Less than 10%) [36]

- Perform local antiseptic cleansing (e.g., chlorhexidine, povidone–iodine);
- Apply dressings: hydrocolloid, hydrogels, or silver-impregnated variants;
- Pain control: paracetamol or Non-Steroidal Anti-Inflammatory Drugs (NSAIDs);
- Avoid routine systemic antibiotics unless clinically indicated;
- Reassess clinically within 48–72 h to confirm healing trajectory.

#### 5.4.2. Moderate to Severe Burns (Deep Grade II and Grade III; TBSA 15–20% in Adults) [37]

- Fluid resuscitation using the Parkland formula:

Fluid (mL) = 4 · bodyweight (kg) · %TBSA

- Administer 50% of calculated volume in the first 8 h, remainder over the next 16 h;
- Only partial- and full-thickness burns count toward TBSA;
- Lactated Ringer’s solution is preferred unless contraindicated.

### 5.5. Limits of the Conventional Therapeutic Approach

Although conventional burn management has advanced substantially, several important limitations persist infection control remains suboptimal despite the use of systemic antibiotics and modern dressings, largely due to the emergence of multidrug-resistant pathogens such as *Pseudomonas aeruginosa* and *Acinetobacter baumannii*, which are further exacerbated by antibiotic-induced dysbiosis and impaired microbial balance [38,39]; wound healing is often delayed, particularly in deep burns requiring grafting, as dermal regeneration is inherently limited, leading to the formation of hypertrophic and fibrotic scars with reduced functional capacity. Systemic complications such as sepsis, renal dysfunction, and profound catabolic states continue to occur in severe burn cases, despite early intervention [40]; in addition, the high costs of surgical procedures, critical care, and prolonged hospitalization place a significant burden on healthcare systems, and finally, psychological recovery and reintegration into social life remain inadequate in many patients, with long-term consequences for mental health and quality of life [41]. These limitations justify exploring complementary biological therapies, including the use of probiotics and their derivatives. Probiotics can influence healing by modulating the microbiome, reducing inflammation, and supporting epithelial regeneration. The integration of these therapies into standard of care could fundamentally transform the paradigm of burn treatment.

## 6. Post-Burning Infections and Microbiotic Imbalances

Burn injuries not only compromise the physical integrity of the skin and deeper tissues but also trigger a complex systemic inflammatory cascade that promotes the onset of severe infections, immune dysregulation, and profound microbial dysbiosis [39,40].

These infectious complications are recognized as the leading cause of morbidity and mortality in burn patients, particularly when early and appropriate treatment is delayed [39]. Understanding the relationship between thermal trauma, microbiome, and host immunity is crucial to developing modern, effective, and sustainable therapeutic strategies.

### 6.1. Pathophysiological Mechanisms and Risk Factors of Post-Burn Infections

After the thermal damage of the skin barrier, the lesion becomes a highly favorable environment for microbial colonization—initially by endogenous cutaneous flora, and subsequently by opportunistic and exogenous pathogens derived from the hospital setting for the patient’s intestinal microbiota. Colonization typically begins within 48 h post trauma and, if improperly controlled, can progress to local infection, cellulitis, necrotizing fasciitis, sepsis, or even multiple organ dysfunction syndrome (MODS) [42]. Key risk factors include impaired local perfusion, tissue ischemia, burn-induced immunosuppression, use of invasive medical devices, and repeated exposure to broad-spectrum antibiotics that disrupt microbial homeostasis [40]. The clinical and immunological efficacy of probiotics in burn patients has been increasingly recognized, particularly in modulating inflammatory responses and enhancing barrier function. Infections are typically indicated by wound discoloration, malodor, peripheral necrosis, bullae, or hemorrhagic lesions, often accompanied by systemic signs such as fever, tachycardia, hypotension, or altered mental status. Definitive diagnosis requires the integration of clinical assessment with microbiological data, including wound swabs, blood cultures, and molecular techniques such as PCR. However, interpretation may be challenging due to the persistent systemic inflammatory response inherent to burn injuries [38].

### 6.2. Post-Burn Sepsis and Multiorgan Dysfunction

**Figure 3:**
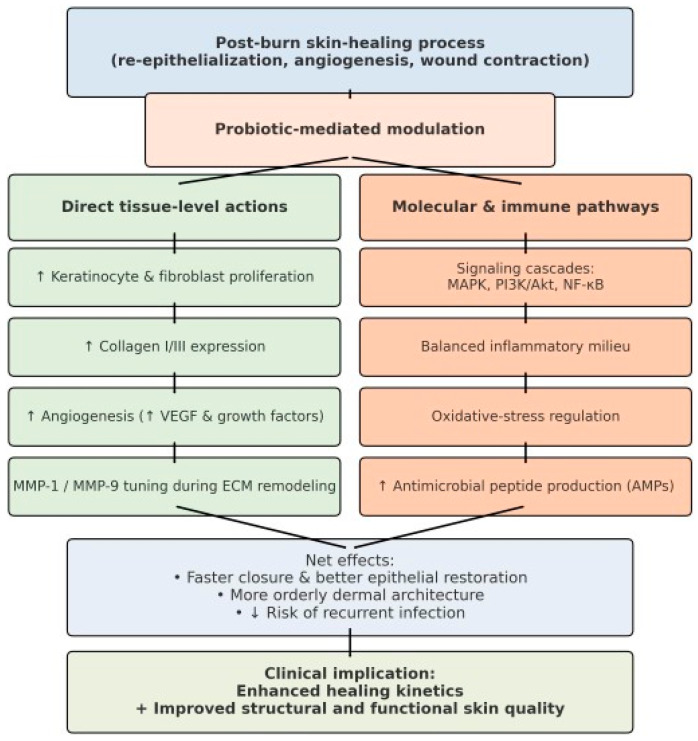
Flowchart of systemic responses after extensive burns.

Sepsis is a significant and frequent complication associated with extensive burn injuries, representing a primary cause of mortality in burn intensive care units [38]. The incidence of sepsis among burn patients with a total body surface area (TBSA) exceeding 30% is reported to surpass 40%, with a mortality rate that may exceed 50%, influenced by factors such as age, comorbidity, and timing of appropriate antimicrobial intervention [38]. The systemic inflammatory response triggered by severe thermal injury compromises the intestinal barrier, facilitating bacterial translocation, activating the complement system, and prompting the release of pro-inflammatory cytokines, including IL-6, IL-1, and IL-18 [43]. Subsequently, this heightened inflammatory state is frequently followed by profound immunosuppression, characterized by elevated IL-10 levels and decreased activation of T-cells and NK cells [11]. This dynamic alternation between hyperinflammation and immunosuppression explains the unpredictable clinical course of burn patients, who may rapidly progress to fulminant sepsis even without a clearly identifiable infectious source (Figure 3). The management of post-burn sepsis involves continuous intensive monitoring, including lactate, procalcitonin, and blood gas analyses; prompt initiation of empirical antibiotic therapy followed by rapid adjustment based on culture and susceptibility testing; aggressive surgical debridement of infected sites; meticulous organ-supportive care, such as mechanical ventilation and renal replacement therapy; immunomodulatory strategies; and intensive nutritional support.

### 6.3. Intestinal and Cutaneous Microbiota in the Context of Burns

The human microbiome, composed of trillions of bacteria, viruses, and fungi, performs essential functions in maintaining local and systemic homeostasis. At the intestinal level, it regulates digestion, mucosal integrity, immune response, and the epithelial barrier. At the cutaneous level, the microbiome interacts with epithelial cells and the innate immune system, producing antimicrobial peptides and maintaining a state of “immunological vigilance”. Severe thermal trauma profoundly alters this microbial ecology. Metagenomic studies [44] have shown that in the first 48–72 h after the trauma, there is

- A significant reduction in beneficial commensal species (*Bifidobacterium*, *Lactobacillus*, *Faecalibacterium prausnitzii*);
- An increase in opportunistic pathogenic bacteria (*Enterobacteriaceae*, *Clostridioides difficile*, *Pseudomonas aeruginosa*);
- An increase in intestinal permeability accompanied by impaired intercellular cohesion, characterized by the downregulation of tight-junction proteins such as zonulin and occludin;
- An increase in the formation of the disease cutaneous dysbiosis with alteration of antimicrobial lipid production and inhibition of keratinocyte regeneration.

This systemic dysbiosis plays a central role in maintaining the state of chronic inflammation, compromising the immune response, and delaying wound healing.

### 6.4. The Impact of Antibiotics on the Microbiome

Antibiotics remain indispensable in the treatment of post-burn infections; nevertheless, repeated and often empirical exposure favors the selection of multidrug-resistant organisms and induces major disturbances of the intestinal and cutaneous microbiome. Broad-spectrum regimens (carbapenems, cephalosporins, fluoroquinolones) reduce microbial diversity and weaken colonization resistance, facilitating overgrowth by fungi, MDR Gram-negative bacteria, and toxigenic *Clostridioides difficile* [39,44]. These adverse effects underpin the growing interest in adjuvant microbiome-based strategies—probiotics, prebiotics, synbiotics, and postbiotics—to restore eubiosis, modulate immune responses, and potentially reduce antimicrobial requirements [14,45].

### 6.5. Pathogens Involved in Nosocomial Infections

In most burn units across Europe and the United States, the predominant pathogens isolated from wounds, blood cultures, and respiratory secretions are *Pseudomonas aeruginosa*, *Acinetobacter baumannii* (often pan-resistant with robust biofilm formation), carbapenems-producing *Klebsiella pneumoniae*, methicillin-resistant *Staphylococcus aureus* (MRSA), and yeasts such as *Candida albicans*; reports increasingly note *Candida auris* as an ICU threat [39,44]. These infections are frequently refractory, relapse-prone, and associated with longer hospital stays, higher costs, and increased mortality, particularly when driven by MDR Gram-negative bacilli [45]. Accordingly, infection control in burn care can no longer rely exclusively on antibiotics; it requires a multimodal, integrative strategy that couples pathogen-directed therapy with biofilm-disrupting measures, restoration of microbial homeostasis (probiotics, prebiotics, synbiotics/postbiotics), and reinforcement of host immune defenses.

### 6.6. The Need for Complementary Therapeutic Strategies

Given the burden and severity of post-burn infections, conventional therapy should be complemented by preventive, immunomodulatory, and microbiome-centered strategies. Interventions that restore intestinal and cutaneous microbial homeostasis—particularly probiotic therapy—demonstrate potential to curb bacterial translocation, lower rates of sepsis and ventilator-associated pneumonia, and modulate host immunity (e.g., increased sIgA, IL-10, and TGF), while concurrently promoting epithelial regeneration and angiogenesis [46]. Collectively, preclinical and clinical evidence indicates that probiotics can serve as an effective adjunct within integrated burn-care protocols, contributing both to infection prevention and to accelerated wound healing [47].

## 7. The Role of Microbiome and Probiotics in Post-Burn Regeneration

The functional and aesthetic restoration of burn-injured skin is orchestrated by complex cellular and molecular pathways that depend on intact immunity, adequate metabolic resources, and balanced host microbiome. Over the past two decades, microbiome research has demonstrated a close interdependence between commensal flora and tissue repair capacity in both acute and chronic wounds [2]. In burn trauma, microbial dysbiosis delays healing, increases infection susceptibility, and is predisposed to pathological scarring. Against this backdrop, probiotics have emerged as a promising adjunctive therapy—capable of modulating local and systemic immunity while actively promoting re-epithelialization, angiogenesis, and extracellular matrix remodeling [47,48].

### 7.1. The Cutaneous Microbiome and Epithelial Regeneration

The cutaneous microbiome comprises a diverse consortium of bacteria, fungi, and viruses—predominantly *Staphylococcus*, *Corynebacterium*, *Cutibacterium* (formerly *Propionibacterium*), and *Micrococcus*—that communicate with keratinocytes, Langerhans cells, and resident T-cells to sustain anti-inflammatory homeostasis and balanced immune tone; under physiological conditions, these communities promote antimicrobial peptide production (e.g., defensins, cathelicidins), maintain an acidic surface pH, and provide colonization resistance against exogenous pathogens [41]. Thermal injury disrupts this symbiosis by destroying epithelial structures and adnexal glands, causing habitat loss, ecological imbalance, and a shift toward pathogenic colonization with pro-inflammatory signaling that delays repair. Emerging evidence indicates that restoration of microbial balance—including via probiotic-based interventions—is associated with faster re-epithelialization, more organized dermal architecture, and higher expression of regeneration-related growth factors, supporting their role as adjuncts in burn wound care pathways [49].

### 7.2. Intestinal Microbiota and Remote Interactions

Although burn injury is localized to the skin, it precipitates systemic alterations—most notably disruption of the gut microbiota—that profoundly affect cutaneous repair. The intestine accounts for approximately 70–80% of host immunological activity, and its resident microbiota modulates systemic inflammation, vascular barrier function, and mesenchymal stem cell activity, all of which are critical to wound healing [42]. Emerging evidence defines a gut–skin axis, wherein microbial metabolites—particularly short-chain fatty acids (SCFAs) such as butyrate, propionate, and acetate—directly enhance keratinocyte migration and proliferation, stimulate angiogenesis, and promote collagen synthesis [50]. In burn models, the administration of probiotics that elevate SCFA production has been shown to attenuate systemic inflammation, reduce bacterial translocation, and accelerate re-epithelialization [51].

### 7.3. Experimental and Clinical Evidence on the Effects of Probiotics in Burns

In recent years, the progressive accumulation of experimental and clinical data has shown that probiotics can play a complex therapeutic role in the management of burn patients with multiple benefits: accelerating tissue healing, reducing the incidence of infections, mitigating systemic inflammation, and restoring microbiotic homeostasis. These effects are the result of sophisticated interactions between probiotic microorganisms and the immune system, epithelial cells, extracellular matrix, and growth factors involved in skin regeneration. Preclinical studies in animal models and controlled clinical research are increasingly contributing to the substantiation of the use of probiotics in the context of burn pathology.

#### 7.3.1. Preclinical Evidence: Experimental Animal Models

Preclinical models, particularly in rodents and pigs, have been instrumental in elucidating probiotic modulation of the burn response. Across studies, both oral and topical administration attenuates inflammatory signaling, reduces pathogenic colonization, upregulates pro-repair gene programs, and improves scar quality. For example, topical *Lactobacillus plantarum* applied to heat-induced cutaneous lesions in rats has been shown to significantly decrease local bacterial burden and accelerate epithelial thickening, accompanied by reduced IL-6 expression in injured tissues [45,52]. At the cellular level, metabolic extracts from *Lactobacillus reuteri* and *Bifidobacterium* spp. promoted proliferation of human dermal fibroblasts in culture and upregulated COL1A1 and MMP1, indicating direct effects on extracellular matrix remodeling [53]. Collectively, these convergent findings indicate that probiotics—delivered topically or systemically—can beneficially influence all major phases of burn healing, from infection control and immunomodulation to re-epithelialization, angiogenesis, and dermal regeneration.

#### 7.3.2. Clinical Evidence

Although clinical evidence remains moderate in size and methodologically heterogeneous, an increasing number of trials report potential benefits of probiotics in moderate to severe burns. For example, in a randomized controlled trial of 72 patients with sepsis (including burn and other critically ill patients), a synbiotic regimen combining *Bifidobacterium breve*, *Lactobacillus casei*, galactooligosaccharides, and dietary fiber significantly reduced the incidence of enteritis (6% vs. 31% in controls, *p* < 0.05) and ventilator-associated pneumonia (9% vs. 40%, *p* < 0.05), while also shortening the duration of antibiotic therapy (average 8.4 vs. 15.1 days) [54,55]. However, these findings should be interpreted with caution: the sample size was limited, the study population included mixed ICU patients (not exclusively burns), and probiotic formulations (strains, doses, and combinations) vary substantially across trials. Syntheses of the literature indicate that probiotic or synbiotic supplementation can lower infectious complications in burn/ICU settings without a consistent signal of serious adverse events, yet safety concerns such as the risk of bacterial translocation and fungemia remain relevant, particularly in severely immunocompromised patients. Overall, while probiotics appear safe, generally well tolerated, and potentially beneficial in high-risk burn patients with dysbiosis, the evidence base is still too heterogeneous to support strong clinical recommendations.

### 7.4. Probiotics Administration

The integration of probiotics in burn therapy requires a careful and rigorous approach that considers the species and strain used, the administered dose, route of administration, duration of treatment, as well as the immunological status of the patient. Although numerous preclinical and clinical studies have demonstrated the safety and efficacy of probiotics, their use in the context of burn patients—often immunocompromised and exposed to multidrug-resistant colonization—should be carefully evaluated and individualized.

#### 7.4.1. Pharmaceutical Forms and Routes of Administration

Probiotics may be delivered via several routes, each characterized by distinct pharmacodynamic features and clinical implications. Oral administration is most prevalent and targets the intestinal microbiota directly. Typical dosage forms include gastro-resistant capsules (e.g., *Lactobacillus casei*, *Bifidobacterium* spp.), sachets containing lyophilized cultures (e.g., *Saccharomyces boulardii*), and synbiotic preparations combining probiotics with prebiotics such as inulin or fructooligosaccharides [56]. Topical administration—such as creams, gels, ointments, or bioactive dressings impregnated with probiotic cultures—is an emerging approach in the management of burn wounds, enabling direct contact with the lesion, localized antimicrobial activity, and potential stimulation of dermal regeneration [57]. Enteral administration via feeding tubes is frequently employed in mechanically ventilated or unconscious patients; in this setting, verification of compatibility between probiotic products and enteral nutrition formulas is essential to preserve microbial viability.

#### 7.4.2. Probiotics in Burn Care: Minimum Effective Doses

The minimum effective dose of probiotics (as shown in [Table 1]) is ≥10^9^ colony forming units (CFU) per day, while high-quality trials typically use 10^9^–10^11^ CFU/day, depending on strain and disease severity. Examples: *Lactobacillus rhamnosus* GG, 1–2 × 10^10^ CFU/day; *Saccharomyces boulardii*, 250–500 mg/day (≈5–10 × 10^9^ CFU); *Bifidobacterium longum*, 10^9^ CFU/day; synbiotic formulas, 10^9^–10^11^ CFU/day combined with 2–4 g/day prebiotics, e.g., inulin or fructooligosaccharides). The usual treatment duration is 7–21 days, tailored to the objective (infection prophylaxis, microbiota restoration, or support of skin regeneration). In critically burned patients, early initiation within 24–48 h of admission has been associated with reduced intestinal colonization by multidrug-resistant organisms [56,58].

#### 7.4.3. Co-Administration of Probiotics and Antibiotics

The stability of probiotics in the gastric acid environment and their sensitivity to antibiotics and heat are critical factors for therapeutic effectiveness. Therefore: co-administration with antibiotics should be carried out at least 2–3 h apart to avoid inactivation of strains; probiotic formulations should be kept under specific temperature conditions (4–8 °C for live lyophilized strains); interactions with anti-secretory drugs (PPI) may increase the bioavailability of probiotics by reducing gastric acidity [59].

### 7.5. Current Challenges and Future Prospects

Despite the obvious benefits, the standardized integration of probiotics into burn treatment protocols faces several barriers:

- The lack of validated clinical guidelines explicitly recommending certain strains;
- Compositional variability of commercial probiotic products without strict control standards;
- The need for large-sample randomized multicenter studies to validate clinical efficiency and long-term safety.

In the long run, probiotic biotechnology is advancing towards the development of personalized probiotics, tailored to the individual microbiome.

## 8. Synthesis of the Mechanisms of Action of Probiotics in Post-Burn Regeneration

Probiotics exert several beneficial effects in the context of burn injuries, through a multitude of cellular, molecular, and microbiological mechanisms. These mechanisms do not act in isolation, but interact in a complex and synergistic way, influencing both local wound healing and systemic homeostasis. The following is an integrative synthesis of these mechanisms, structured on major functional dimensions:

- Immunomodulation;
- Tissue regeneration;
- Antimicrobial effect;
- Microbiotic balance and epithelial protection.

### 8.1. Modulation of Local and Systemic Immune Response

Severe thermal trauma triggers a broad systemic inflammatory response marked by massive production of pro-inflammatory cytokines (IL-6), oxidative stress, and complement activation. In parallel, a phase of immunoparalysis follows, characterized by increased IL-10, decreased MHC-II expression on monocytes, and suppression of T-cell function [31,60]. Probiotics influence this immunological balance by stimulating production of anti-inflammatory cytokines (IL-10) with concomitant reductions in pro-inflammatory markers, activating tolerogenic dendritic cells and regulating T-cell polarization toward a Treg phenotype at the expense of Th1/Th17, stimulating secretory IgA production in the intestinal mucosa, thereby strengthening mucosal immunity; and regulating co-stimulatory molecule expression while restoring monocyte and macrophage function (Table 2) [60]. Thus, probiotics help rebalance the immune response, attenuating excessive inflammation and restoring the functional immunity required for tissue regeneration.

### 8.2. Tissue Regeneration and Angiogenesis

The post-burn skin-healing process involves re-epithelialization, angiogenesis, and wound contraction [61]. Probiotics can modulate these phases by stimulating keratinocyte and fibroblast proliferation; increasing collagen I/III expression; enhancing angiogenesis through up-regulation of VEGF and other growth factors; and tuning matrix metalloproteinases (e.g., MMP-1, MMP-9) during ECM remodeling and cell migration [62]. The net effect is faster closure with better epithelial restoration, more orderly dermal architecture, and a lower risk of recurrent infection [63]. This probiotic-mediated modulation of wound repair reflects a multifaceted interaction between host cells, microbial metabolites, and immune signaling pathways. By influencing key molecular cascades such as MAPK, 582 PI3K/Akt, and NF-κB, probiotics not only enhance cellular proliferation and migration but also maintain a balanced inflammatory milieu conducive to tissue regeneration. Moreover, their ability to regulate oxidative stress and promote antimicrobial peptide production provides an additional layer of protection in the vulnerable post-burn environment. Collectively, these effects highlight the therapeutic potential of probiotics as adjuvant agents in burn care, offering a strategy to improve healing kinetics while simultaneously enhancing the structural and functional quality of the regenerated skin.

### 8.3. Direct and Indirect Antimicrobial Effect

One of the most important properties of probiotics in the context of burns is the ability to reduce local and systemic pathogenic colonization by competition for nutrients and adhesion sites on epithelial cells and extracellular matrix; production of antimicrobial substances such as bacteriocins (e.g., plantaricin, acidolin), hydrogen peroxide, short-chain fatty acids (SCFAs), lactic and acetic acids; modification of local pH—the acidification of the environment inhibits opportunistic Gram-negative bacteria; and disruption of pathogen biofilm formation by inhibiting expression of adhesion/coagulation genes (e.g., lasI, rhlR in *Pseudomonas aeruginosa*) [61,62].

### 8.4. Restoration of the Microbiotic Balance (Eubiosis)

Intestinal and cutaneous dysbiosis generated by burn injury is associated with a reduction in commensal species (e.g., *Faecalibacterium prausnitzii*, *Akkermansia muciniphila*), increased *Enterobacteriaceae* and *Clostridioides difficile* and altered epithelial barrier function. Probiotics modulate these dysfunctions by restoring bacterial diversity with growth of SCFA-producing strains, stimulating tight-junction protein expression (occludin, claudin-1) to reduce permeability and translocation, and interfering with pathogen quorum sensing to lower virulence and toxin release. This re-establishment of eubiosis correlates with reduced circulating endotoxin (LPS), improved systemic immunity, and faster, more effective wound healing [64,65].

## 9. Current Clinical Considerations and Future Therapeutic Perspectives

The integration of probiotics into therapeutic protocols for patients with severe burns is an emerging frontier in regenerative medicine and management of nosocomial infections. Although preclinical evidence is compelling and clinical trials offer promising results, there are many issues that need to be clarified before probiotics become an integral and standardized part of treatment for burn patients. This section examines current clinical implications, methodological challenges, associated risks, and future priority research directions.

### 9.1. Indications and Profiles of Patients’ Candidates for Probiotic Therapy

Based on the available studies, the following categories of patients could benefit from taking probiotics in burn treatment:

- Patients with extensive burns (more than 15–20% TBSA) at increased risk of systemic infections and intestinal dysbiosis;
- Mechanically ventilated patients, enteral nutrients, with prolonged use of broad-spectrum antibiotics;
- Patients with leaky gut syndrome and markers of bacterial translocation;
- Patients with recurrent wound infections, colonization with *Clostridium difficile*;
- Patients in the subacute recovery phase, aimed at accelerating healing and reducing chronic inflammation.

In these cases, probiotics can be used either prophylactically to reduce the risk of infection and immune imbalance, or adjuvant therapy to complement antimicrobial and supportive treatment.

### 9.2. Current Limitations and Challenges of Clinical Implementation

Biotechnological research in the field of probiotics has experienced a spectacular evolution in recent years, proposing new therapeutic directions adapted to the complexity of burn pathology:

- Next-generation probiotics include fewer known strains with strong immunomodulatory effects, such as *Faecalibacterium prausnitzii* and *Akkermansia muciniphila*, with special applicability in chronic inflammation and epithelial regeneration [52].
- Postbiotics—bioactive substances produced by probiotics (SCFAs, peptides, polysaccharides, enzymes) administered directly without live microorganisms—offer increased stability and a higher safety profile [65].
- Synergistic bacterial-consortium therapy uses combinations of strains chosen for complementary action on immune response and tissue regeneration [66].
- Microbiome-personalized therapy tailors probiotics to the individual patient’s microbiotic profile determined by high-resolution metagenomics.
- Smart probiotic dressings—wound coverings impregnated with beneficial bacteria—create a microclimate favorable to regeneration while inhibiting pathogenic colonization [66].

An exploratory, single-center study (n = 80) suggests that topical application of a live *Lactobacillus plantarum* culture may offer a pragmatic, “silver-sparing” option in selected burn wounds, with performance comparable to silver sulfadiazine across several endpoints and possible advantages in infected, delayed third-degree injuries. In delayed second-degree burns (infected at baseline), the proportion of patients achieving the composite target—reduction in bacterial load below 105 per gram of tissue together with granulation and subsequent healing—was 71% (10/14) with *L. plantarum* versus 73% (11/15) with silver sulfadiazine. In early third-degree burns (clinically non-infected), granulation suitable for grafting occurred in 75% (10/12) versus 84% (11/13); graft take was 90% (9/10) versus 90% (10/11); and complete healing was 75% (9/12) versus 77% (10/13) for *L. plantarum* and control, respectively. In delayed third-degree burns (infected), *L. plantarum showed* favorable signals: bacterial load reduction with granulation in 83% (10/12) versus 71% (10/14), identical graft takes (90%, 9/10 in both arms), and higher complete healing 75% (9/12) versus 64% (9/14) (Table 3). No invasive infections were detected during follow-up and *L. plantarum* was not recoverable from wounds after treatment, supporting short-term safety and lack of persistence. Although underpowered (subgroups, 12–15 patients) and exploratory—with differences in co-interventions and outcomes reported as proportions—the dataset outlines concrete clinical opportunities to test topical *L. plantarum* in infected or delayed burns within adequately powered, multicenter trials and to evaluate integration with advanced dressings that target biofilm-rich wounds [67].

Another randomized, double-blind, placebo-controlled clinical trial in 40 hospitalized children with thermal burns (20–50% total body surface area; admission within 24 h) evaluated an oral probiotic adjunct to standard care. The intervention was Lacteol Forte, a two-strain preparation containing Lactobacillus fermentum and *Lactobacillus delbrueckii*, administered twice daily; each sachet contained 1010 live bacteria (daily total 2 × 1010), dissolved in 50 mL water and continued for the duration of hospitalization. The comparator was a matched placebo (starch). Both arms received identical background management, including daily 1% silver sulfadiazine dressings and standardized early enteral nutrition. Compared with placebo, the probiotic arm experienced fewer diarrhea episodes (three vs. nine; 676 *p* = 0.038), fewer patients requiring skin grafting (2/20 vs. 8/20; *p* = 0.028), a shorter length of stay (17.25 ± 0.5 vs. 21.9 ± 2.2 days; *p* = 0.044), and—among patients who did not require grafting—a faster time to complete healing (16.5 ± 0.23 vs. 20.7 ± 0.51 days; *p* = 0.048). Infectious complications were numerically lower but not statistically significant (7 vs. 12; *p* = 0.113). Laboratory readouts on day 14 supported a biological signal (lower C-reactive protein, *p* = 0.032; higher IgA, *p* = 0.033). Safety was acceptable: no deaths, no invasive infections attributed to the intervention, and a higher incidence of flatulence in the probiotic group (*p* = 0.006). Although limited by its single-center design and small sample size in a pediatric population, the study demonstrates that oral preparation delivering 10^10^ live bacteria per dose (two doses daily) can be feasibly integrated into burn care and may confer clinically relevant benefits that warrant confirmation in larger multicenter trials with standardized endpoints [68]. From a methodological standpoint, the restricted cohort size substantially limits statistical power, raising concerns regarding type II error and the stability of observed effect sizes, while the absence of randomization or blinding introduces potential risks of selection bias and performance bias. Moreover, the exclusive focus on a pediatric population constrains generalizability, as age-related immunological differences and variations in gut microbiota composition may influence the host–probiotic interaction in ways that are not directly transferable to adult burn patients. Nevertheless, the study contributes valuable preliminary insights into the safety, tolerability, and practical implementation of high-dose probiotic supplementation in an acutely vulnerable cohort, suggesting that such an intervention may modulate both local wound repair processes and systemic immune responses. To build on these findings (presented in [Table 4] as well), future investigations should employ rigorously designed, adequately powered multicenter randomized controlled trials that incorporate standardized and clinically meaningful outcome measures—including infection rates, length of hospitalization, incidence of sepsis, long-term functional recovery, and quality-of-life indices—while also addressing potential confounding variables such as concomitant antimicrobial therapies, nutritional status, and burn severity. Only through such methodologically robust approaches can the true therapeutic potential of probiotics in pediatric burn care be comprehensively evaluated and translated into evidence-based clinical practice.

Clinical evidence, although still limited, indicates that probiotics may offer clinically relevant benefits in the management of burn patients by influencing both systemic immune responses and local wound healing dynamics. Oral supplementation, particularly in pediatric populations, has been shown to be feasible, safe, and well tolerated, with preliminary signals suggesting a reduction in secondary infections and attenuation of intestinal dysbiosis. Such findings are noteworthy given the high vulnerability of pediatric burn patients, where immune immaturity and the risk of infection often complicate recovery. In adult cohorts, topical administration of *Lactobacillus plantarum* has produced outcomes (represented in [Table 5]) that are comparable or even superior to conventional silver-based treatments, including a decrease in bacterial burden, enhanced granulation, and higher rates of complete wound closure, even under conditions of delayed healing or infection. Taken together, these studies point toward a potential adjuvant role of probiotics in burn care, with effects that may extend beyond simple antimicrobial activity to include modulation of host immune pathways, reinforcement of epithelial integrity, and acceleration of tissue regeneration. Nonetheless, the existing evidence base is restricted by small sample sizes, single-center designs, and heterogeneity in treatment protocols. Future multicenter, randomized controlled trials with standardized endpoints such as infection incidence, length of hospitalization, graft success, and long-term functional recovery are urgently required to validate these promising results and establish evidence-based guidelines for the integration of probiotics into routine burn management.

### 9.3. Future Research Needs and Recommendations

To definitively validate the role of probiotics in the treatment of burns and support their integration into international guidelines, the following are required:

- Large, multicenter randomized trials. Double-blind, placebo-controlled comparisons of well-defined probiotic (or synbiotic/postbiotic) formulations, powered for burn-specific primary outcomes (wound infection, graft take, time to closure/epithelialization) and key secondary outcomes (antibiotic days, length of stay, bacteremia/ventilator-associated pneumonia, organ-support-free days, patient-reported symptoms). Include pre-specified strata by burn severity (e.g., %TBSA, inhalation injury) and by route (topical vs. enteral).
- Integrated multi-omics. Longitudinal metagenomic, proteomic, and transcriptomic profiling of wound and gut samples to map effects on pathogen load, biofilm signatures, host inflammatory pathways, and tissue-repair programs using standardized sampling windows (baseline, day 3–5, pre-graft, post-graft, discharge).
- Pharmacokinetic/pharmacodynamic and formulation studies. Head-to-head evaluations of strains, doses and timing (early initiation ≤ 48 h vs. delayed), and routes (topical dressings vs. enteral) with measurement of viability at administration, persistence/clearance, local concentrations in the wound, and host-response markers; optimize delivery systems (e.g., hydrogels/smart dressings) for stability and controlled release.
- Safety surveillance. Burn-specific risk management with independent monitoring committees: systematic blood/wound cultures for potential translocation, adjudicated adverse events (including central line-associated events), whole-genome screening of candidate strains for resistance and virulence genes, and multicenter registries to detect rare harms.
- Economic evaluation. Prospective cost-effectiveness and budget-impact analyses capturing length of hospitalization, operating-room episodes, antibiotic consumption, readmissions, and quality-of-life metrics; include scenario and equity analyses relevant to low- and middle-income burn centers.
- Standardization and reporting. Adoption of a core outcome set for burn–microbiome trials, preregistered protocols, CONSORT-compliant reporting, clear strain identity (deposition numbers), explicit dose notation and viability both at end of shelf life and at administration, and transparent documentation of co-interventions (debridement, dressings, antibiotics).
- Regulatory translation. Early engagement with regulators on classification (live biotherapeutic vs. combination product), manufacturing quality, labeling and post-marketing pharmacovigilance to enable guideline inclusion once efficacy and safety are established.

*Synthesis*. This agenda shifts the field from mechanism-heavy preclinical work to decision grade clinical evidence. A practical pathway is a staged program in which phase I/II studies establish dose, timing, and route while embedding multi-omics and safety assays, followed by adequately powered multicentre phase III trials with standardized outcomes and economic endpoints. If benefit and safety are confirmed—particularly in severe, infection-prone burns—probiotics can progress from exploratory adjuncts to evidence-based components of integrated burn care, aligned with antimicrobial stewardship and personalized-medicine goals.

## 10. Conclusions

Probiotics and synbiotics are promising adjuncts in severe burn care, but their value will be established by targeted, patient-centered research rather than uniform products. Upcoming trials should be personalized at the level of individual strains using integrated genomic and metabolic profiling alongside clinical stratification by percentage of total body surface area burned, presence of inhalation injury, and immune status. Early, adaptive studies should map dose, timing (early initiation within 24–48 h versus later), and route of delivery (enteral versus topical to the wound) before larger multicenter trials proceed. Integration with smart dressings—microencapsulated live biotherapeutics or postbiotic payloads embedded in hydrogels that respond to local acidity or reactive oxygen species, with built-in biosensors—could enable on-demand anti-infective and anti-biofilm action; platform trial designs can efficiently evaluate combinations that pair probiotic therapy with smart dressing technologies and antibiotic regimens. Translation will depend on manufacturing to rigorous pharmaceutical-quality standards, full genomic safety screening, verified viable cell counts at the time of administration, and registries capable of detecting rare harms such as fungemia or events related to central lines. Regulators should clarify pathways for device–drug combination products, have labeling that states per-strain viable cell counts and viability at the end of shelf life, and data governance for selection guided by molecular profiling. With standardized outcomes and robust safety oversight, probiotics could move from exploratory use to a defined adjunct in burn care.

## Figures and Tables

**Table 1:** Probiotic/synbiotic dosing.

Product/Strain	Typical Daily Dose	Range	Prebiotic	Key Notes
Minimum effective (general)	≥ 1 × 109 CFU/day	109–1011 CFU/day	—	severity; in burn care start within 24–48 h.
*Lactobacillus rhamnosus* GG (LGG)	1–2 × 1010 CFU/day	109–2 × 1010 CFU/day	Optional (inulin)	Well studied; prefer gastro-resistant oral forms.
*Saccharomyces boulardii*	250–500 mg/day (≈ 5–10 × 109 CFU)	5 × 109–1 × 1010 CFU/day	Not required	Avoid in severe immunosuppression or with central venous catheters.
*Bifidobacterium longum*	1 × 109 CFU/day	109–1010 CFU/day	Often inulin	Synbiotic pairing may enhance engraftment.
Synbiotic formulas	109–1011 CFU/day	—	2–4 g/day (inulin)	Match prebiotic to strain; verify

Sources: Adapted from Baral et al., 2021 [56]. Clinical references: As reported in clinical burn care studies [56,58], early initiation of probiotics (within 24–48 h post-admission) has been associated with reduced intestinal colonization by multidrug-resistant organisms and improved microbiota restoration.

**Table 2:** Immune dysregulation after severe burns and potential mechanisms by which probiotics contribute to immune rebalancing.

Severe Burn Injury/Immune Dysregulation	Probiotic-Mediated Mechanisms	Restored Immune Balance
**Hyper-inflammatory phase** - IL-6 ↑ - Oxidative stress - Complement activation	- Reduction in pro-inflammatory cytokines (IL-6, TNF-*α*) - Induction of anti-inflammatory cytokines (IL-10)	- Attenuation of excessive systemic inflammation
**Immunoparalysis phase** - IL-10 ↑ - MHC-II on monocytes ↓ - T-cell suppression	- Activation of tolerogenic dendritic cells - T-cell polarization toward Treg (↓Th1/Th17) - Regulation of co-stimulatory molecules - Restoration of monocyte/macrophage function	- Rebalanced innate and adaptive responses - Functional immune recovery
**Barrier dysfunction** - Impaired mucosal immunity	- Increased secretory IgA production - Strengthening of mucosal barrier	- Improved barrier integrity - Enhanced resistance to infection

Source: Adapted from Osuka et al., 2024, [60]. **Clinical note:** Probiotic-mediated mechanisms have been described to attenuate hyperinflammation, counteract immunoparalysis, and restore barrier integrity, thereby contributing to immune rebalancing after severe burn injury.

**Table 3:** Outcomes by subgroup: topical *Lactobacillus plantarum* vs. 1% silver sulfadiazine.

Subgroup	Outcome	Probiotic (n/N, %)	Control (n/N, %)	∆ pp (95% CI)	RR (95% CI)
Delayed II° (infected)	Composite *	10/14 (71.4)	11/15 (73.3)	−1.9 (−34.5–30.7)	0.97 (0.62–1.53)
Early III° (non-infected)	Granulation for grafting	10/12 (83.3)	11/13 (84.6)	−1.3 (−30.1–27.5)	0.98 (0.70–1.39)
Early III° (non-infected)	Graft take	9/10 (90.0)	10/11 (90.9)	−0.9 (−26.1–24.3)	0.99 (0.75–1.31)
Early III° (non-infected)	Complete healing	9/12 (75.0)	10/13 (76.9)	−1.9 (−35.5–31.6)	0.97 (0.63–1.52)
Delayed III° (infected)	Composite †	10/12 (83.3)	10/14 (71.4)	+11.9 (−19.8–43.6)	1.17 (0.77–1.77)
Delayed III° (infected)	Graft take	9/10 (90.0)	9/10 (90.0)	0.0 (−26.3–26.3)	1.00 (0.75–1.34)
Delayed III° (infected)	Complete healing	9/12 (75.0)	9/14 (64.3)	+10.7 (−24.4–45.8)	1.17 (0.70–1.94)
*Notes:* ∆ pp = absolute difference in percentage points (probiotic minus control); RR = risk ratio. * Composite = bacterial load *<* 10^5^/g + granulation + healing. † Composite = bacterial load *<* 10^5^/g + granulation. Subgroup sizes ∼12–15 patients; Source: Peral et al. [67].					

**Clinical note:** Subgroup analyses (12–15 patients) suggested favorable effects of *L. plantarum* on bacterial load reduction, granulation, and complete healing, compared with silver sulfadiazine. These results, while promising, remain exploratory due to limited power and sample size.

**Table 4:** Key points on the gut–skin axis and probiotics in burns (brief).

Key Point	Brief Mechanism/Effect	Ref.
Burn injury triggers systemic alterations with gut dysbiosis	Systemic changes that influence cutaneous	[43]
Intestine hosts ∼70–80% of host immune activity	Microbiota modulates systemic inflammation, vascular barrier function, and mesenchymal stem cells	[43]
Gut–skin axis	Microbial metabolites link gut signals to skin healing responses	[52]
SCFAs (butyrate, propionate, acetate)	Keratinocyte migration/proliferation ↑; Angiogenesis↑ collagen sinthesis ↑	[52]
Probiotics elevating SCFA in burn models	Systemic inflammation ↓; bacterial translocation; re-epithelization↑	[53]

Source: Adapted from El-Ghazely et al. [68]. **Clinical note:** The study highlights the gut–skin axis as a key mediator in burn recovery, illustrating how probiotic-induced modulation of microbial metabolites (e.g., SCFAs) contributes to attenuating systemic inflammation, enhancing keratinocyte activity, and supporting re-epithelialization in pediatric burn patients.

**Table 5:** Summary of clinical studies evaluating probiotics in burn patients.

Burn Severity/Population	Intervention	Main Outcomes
Pediatric moderate–severe burns	Oral probiotic, 10^10^ CFU/dose, twice daily	Feasible integration into standard burn care; safe and well tolerated; potential reduction in infections and intestinal dysbiosis. ^1^
Pediatric burns (n = 80), single-center	Probiotics as adjuvant therapy	Indications of immunomodulatory effects; limited by small sample size and lack of multicenter validation. ^1^
Adults with second- and third-degree burns	Topical *Lactobacillus* *plantarum* vs. 1% silver sulfadiazine	Comparable or superior efficacy: lower bacterial load, improved granulation, complete healing in 75–77% of cases; no invasive infections; well tolerated. ^2^
Infected, delayed-healing burns	Topical *L. plantarum*	Reduced bacterial colonization; improved granulation (83% vs. 71% in controls); higher complete healing rate (75% vs. 64%). ^2^

Source: Adapted from ^1^ El-Ghazely et al. [68] and ^2^ Peral et al. [67].

## Data Availability

Not applicable.

## Abbreviations

The following abbreviations are used in this manuscript:

WHO	World Health Organization
SIRS	systemic inflammatory response syndrome
*MRSA*	*Methicillin-resistant Staphylococcus aureus*
TBSA	total body surface area
NSAIDs	Non-Steroidal Anti-Inflammatory Drugs
MODS	multiple organ dysfunction syndrome
SCFAs	short-chain fatty acids
CFU	colony forming units
PPI	anti-secretory drugs
MDR	Multi-Drug Resistance
IL-6	Interleukina-6
IL-1	Interleukina-1
IL-10	Interleukina 10
IL-18	Interleukina 18
PCR	Polymerase Chain Reaction
IgA	Immunoglobulin A
TGF	Transforming Growth Factor
COL1A1	Collagen TypeI Alpha 1Chain
MMP1	Matrix Metalloproteinase 1
MMP-9	Matrix Metalloproteinase 9
ECM	Extracellular Matrix
ICU	Intensive Care Unit
Th1	T helper 1
Th 17	T helper 17
VEGF	Vascular Endothelial Growth Factor
MAPK	Mitogen-Activated Protein Kinase
PI3K/Akt	Phosphoinositide 3-Kinase / Protein Kinase B
NF-κB	Nuclear Factor kappa-light-chain-enhancer of activated B cells
LPS	Lipopolysaccharide
